# Exposure of stevia (*Stevia rebaudiana* B.) to silver nanoparticles *in vitro*: transport and accumulation

**DOI:** 10.1038/s41598-019-46828-y

**Published:** 2019-07-17

**Authors:** Celia G. Castro-González, Lino Sánchez-Segura, Fernando C. Gómez-Merino, Jericó J. Bello-Bello

**Affiliations:** 1 Colegio de Postgraduados, Km 348 carr, federal Córdoba-Veracruz, Congregación Manuel León, 94946 Municipio de Amatlán de los Reyes, Veracruz, Mexico; 2 Departamento de Ingeniería Genética, Unidad Irapuato, CINVESTAV-Irapuato, Libramiento Norte Carr, Irapuato-León Km 96, Irapuato, Guanajuato Mexico; 3 CONACYT-Campus Córdoba, Colegio de Postgraduados, Km 348 carr, federal Córdoba-Veracruz, Congregación Manuel León, 94946 Municipio de Amatlán de los Reyes, Veracruz, Mexico

**Keywords:** Plant development, Nanoparticles

## Abstract

The impact of nanotechnology in the field of agricultural sciences creates the need to study in greater detail the effect of products offering nanoparticles for application in plant species of agricultural interest. The objective of this study was to determine the response of stevia (*Stevia rebaudiana* B.) *in vitro* to different concentrations of AgNPs (silver nanoparticles), as well as to characterize and identify their absorption, translocation and accumulation mechanisms. Nodal segments of stevia grown in MS medium supplemented with AgNPs (0,12.5, 25, 50,100 and 200 mg L^−1^) were used. After 30 days of *in vitro* shoot proliferation, the number of shoots per explant, shoot length, chlorophyll content, dry matter content and the metallic silver (Ag) content of the plants were quantified. In addition, characterization, transport and accumulation of silver nanoparticles were performed by microscopic analysis. AgNPs were shown to be present in epidermal stem cells, within vascular bundles and in intermembrane spaces. In leaves, they were observed in ribs and stomata. The current and future use of AgNPs in agricultural sciences opens up the possibility of studying their effects on different plant species.

## Introduction

The use of silver nanoparticles (AgNPs) in biological systems is part of the development of bionanotechnology. This discipline offers alternatives to solve problems related to the production of food crops by inhibiting microbial agents^1–3^. In plants, AgNPs have been used to induce germination, increase crop yields and promote development^4,5^.Some studies have focused on the description of antimicrobial activity and hormesis under *in vitro* conditions, in which development changes and increased biomass production have been observed^6–9^. The hormetic effect is characterized by growth stimulation at low doses and inhibition at high doses^10^. On the other hand, the AgNPs concentration, size, shape, chemical composition, reactivity, type of coating and levels of aggregation may affect plant development^11,12^. However, the absorption, translocation and accumulation mechanisms of AgNPs in plants have been poorly studied and elucidated.

The development of new methods to characterize and identify AgNPs in cells and tissues would contribute to a better understanding of the potential effects during *in vitro* culture of plant tissues. To date, microscopy techniques have been used to study the assimilation and accumulation of NPs in plants under *in vitro* conditions^11,13^. *In vitro* culture is a technique that allows studying the behavior of AgNPs in plants of agricultural interest such as stevia (*Stevia rebaudiana* B.). Stevia cultivation has increased in importance in the food industry in recent years due to its high content of steviosides used as sweeteners^14^. The importance of studying the transport mechanisms and effects that may be generated by AgNPs in plants of commercial interest, will help to determine the effects they could have in the application of sanitizer products intended for agriculture. The objective of this study was to characterize the physicochemical properties of AgNPs formulated as Agrovit, as well as to study the effect of their concentration on the development of stevia (*Stevia rebaudiana* B.) grown *in vitro*.

## Results

### Physicochemical Characteristics of Argovit

**Figure 1:**
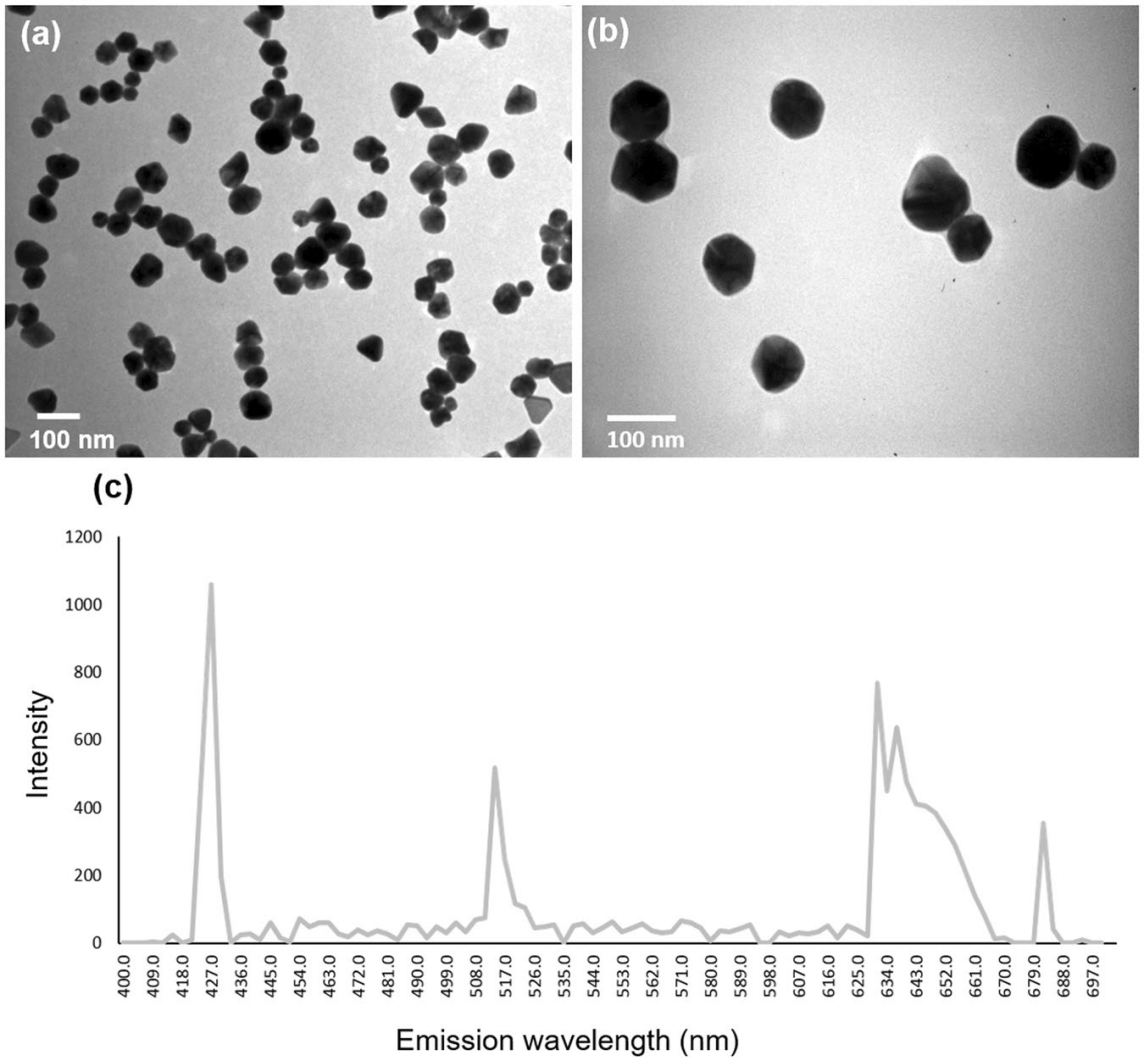
TEM image of AgNPs using different magnifications. (**a**) Bar = 100 nm, (**b**) Bar = 100 nm and (**c**) Fluorescence by multiphoton microscopy.

The physicochemical characteristics of AgNPs are summarized in Table 1. The AgNPs characterized by TEM are shown round, with a 0.82 shape factor (*sf*) and 0.88 roundness. The analysis of the AgNPs dimensions showed average diameters of 35 ± 15 nm, which is silver functionalized with PVP (Fig. 1a,b). The spectral analysis of the AgNPs showed the highest fluorescence at 427 nm (Fig. 1c).

**Table 1:** Physicochemical Characteristics of Argovit.

Properties	Mean
Metallic silver content (% wt.)	1.2
PVP Content (% wt.)	18.8
Form Factor (Spheroid)	0.82
Average diameter of metallic silver particles by TEM data (nm)	35 ± 10
Round	0.88
Size interval of metallic silver particles by TEM (nm)	1 a 80
Zeta potential (mV)	−15
Fluorescence in violet, green and red (nm)	427, 514 and 631

Abbreviations: Ag, silver; PVP, polyvinylpyrolidone; TEM, transmission electron microscopic.

### Effect of AgNPs on *in vitro* shoot proliferation

**Figure 2:**
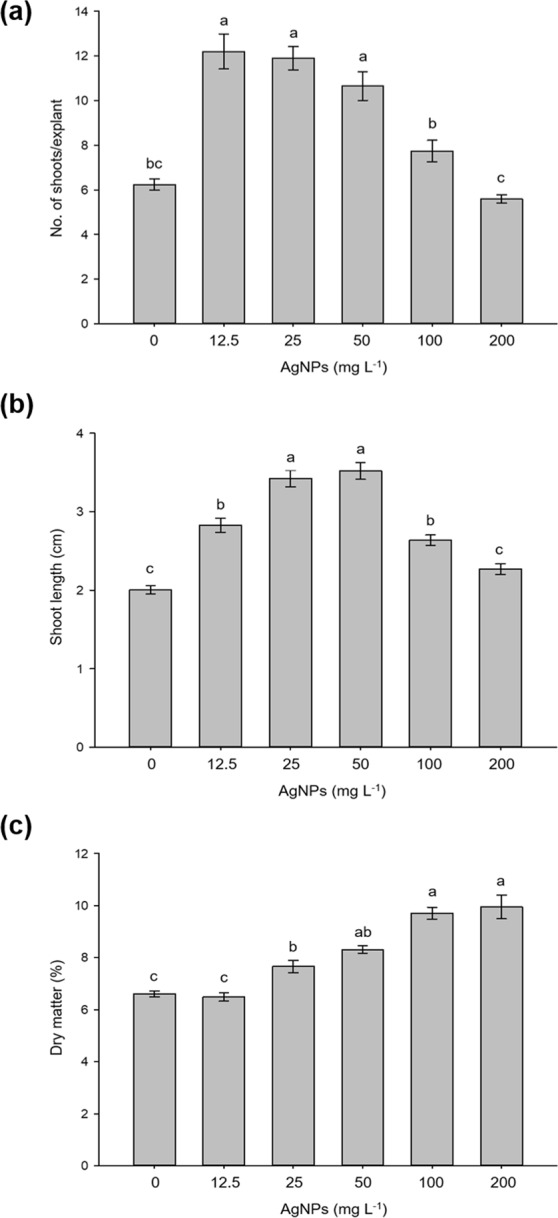
Effect of AgNPs on the *in vitro* regeneration of stevia (*Stevia rebaudiana* B.) (**a**) Number of shoots per explant, (**b**) shoot length and (**c**) dry matter. Mean ± standard error within a bar followed by the same letter are not significantly different according to Tukey’s test at (*P* ≤ 0.05).

**Figure 3:**
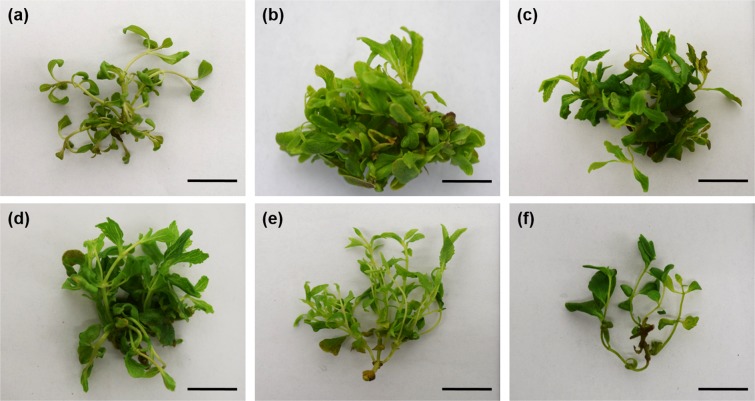
Effect of Silver nanoparticles on *in vitro* multiplication of stevia (*Stevia rebaudiana* B.) after 30 days of culture in Temporary immersion systems (TIB). (**a–f**) 0, 12.5, 25, 50, 100 y 200 mg L^−1^ of Argovit, respectively. *Bar* = 1 cm.

When evaluating the effect of AgNPs on *in vitro* regeneration of stevia, significant differences were observed among the variables evaluated (Fig. 2). Applying 12.5, 25 and 50 mg L^−1^ of AgNPs promoted the highest shoot production and length per explant, while the control treatment and 200 mg L^−1^ of AgNPs showed the lowest shoot production and length (Fig. 3). The dry matter gradually increases from 25 mg L^−1^ of AgNPs, obtaining the highest values with the 100 and 200 mg L^−1^ AgNPs concentrations, with 9.7 and 9.9 dry matter, respectively (Fig. 2c).

### Measurement of silver, magnesium, nitrogen and iron content

Significant differences (p ≤ 0.05) were detected for N, Mg, Fe and Ag contents in Argovit treatments (Table 2). N and Mg accumulated in higher amounts when plants were exposed to 50, 100 and 200 mg L^−1^ of AgNPs. For Fe we detected a depletion of the element in AgNPs treatments in the highest concentrations. The results obtained in the quantification of Ag showed significant differences. Overall, an increase in Ag content in tissues was observed as AgNPs concentrations in the culture medium increased. The lowest Ag content was observed in the control treatment without AgNPs, obtaining 0.13 µg g^−1^ dw (dry weight) of Ag, while the highest Ag content was observed in the treatments with 100 and 200 mg L^−1^ of AgNPs, with 95.23 and 188.16 µg g^−1^ dw of Ag, respectively.

**Table 2:** Effect of AgNPs on N, Mg, Fe and Ag content in stevia (*Stevia rebaudiana* B.) shoots after 30 days of *in vitro* culture.

AgNPs (mg L^−1^)	Elements (µg g^−1^)			
	N	Mg	Fe	Ag
0	38067.33 ± 45.27^c^	882.41 ± 9.47^c^	289.31 ± 4.50^a^	0.13 ± 0.00^e^
12.5	47902.00 ± 73.32^b^	1125.74 ± 8.23^b^	282.46 ± 3.43^a^	8.63 ± 0.03^d^
25	48243.33 ± 422.64^b^	1118.69 ± 3.16^b^	287.71 ± 2.70^a^	9.01 ± 0.007^d^
50	54877.33 ± 28.81^a^	1219.53 ± 0.23^a^	166.51 ± 0.30^b^	21.05 ± 0.02^c^
100	55313.00 ± 6.02^a^	1242.22 ± 0.68^a^	163.32 ± 1.59^b^	95.23 ± 0.02^b^
200	55339.67 ± 31.10^a^	1292.62 ± 11.17^a^	143.28 ± 0.74^c^	188.16 ± 1.65^a^

Means ± standard error within a column followed by the same letter are not significantly different according to Tukey’s test at *p* ≤ 0.05. Elements: *N* total nitrogen, *Mg* magnesium, *Fe* iron and *Ag* silver.

### Effect of silver nanoparticles on chlorophyll contents

**Figure 4:**
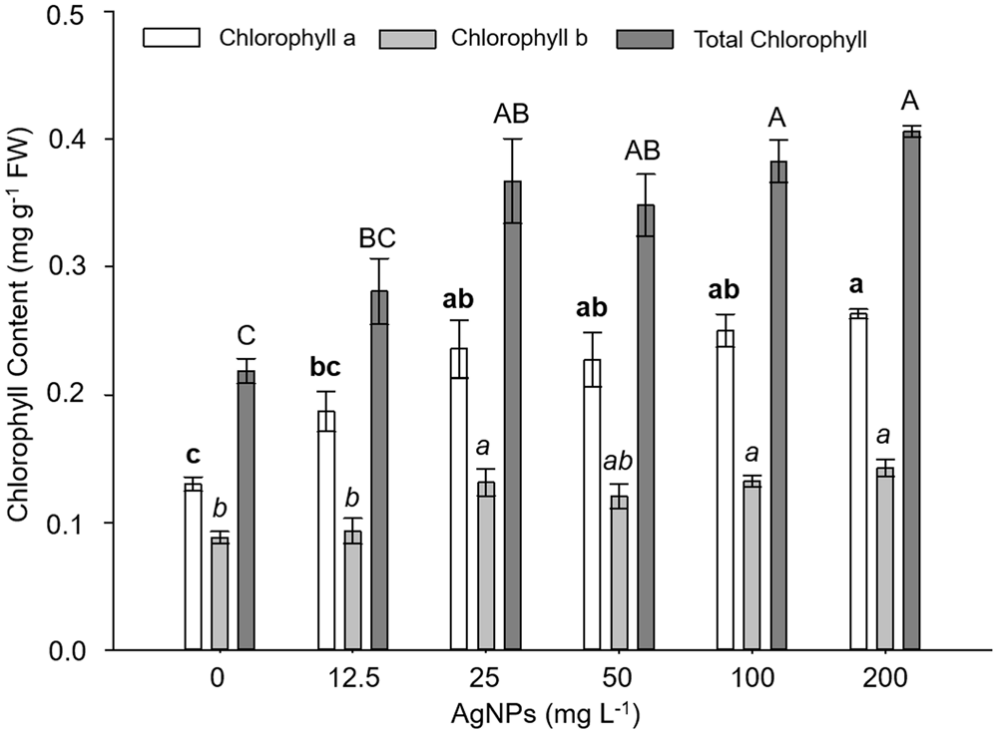
Chlorophyll content after stevia (*Stevia rebaudiana* B.) exposure to different concentrations of Argovit. Mean ± standard error within a bar followed by the *same letter* are not significantly different according to Tukey’s test at (*P* ≤ 0.05).

The chlorophyll a, b and total contents showed significant differences among the AgNPs concentrations evaluated (Fig. 4). In general, an increase in chlorophyll content from 25 mg L^−1^ was observed. Chlorophyll a, b and total contents were lower in the control treatment and at the lowest concentration of AgNPs evaluated (12.5 mg L^1^).

### Detection of silver in stem and leaf cells by fluorescence microscopy

**Figure 5:**
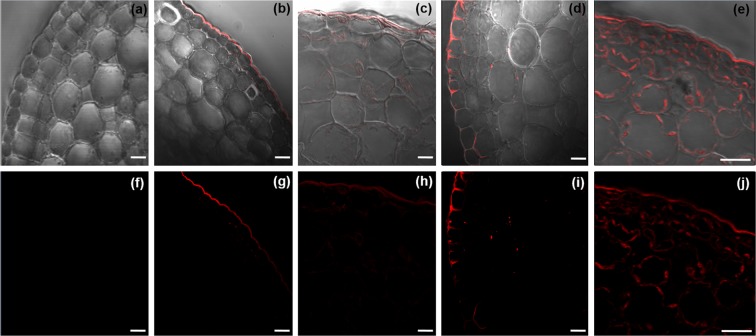
Identification of fluorescence at specific points in epidermis of stevia stems at 0, 25, 50, 100 and 200 mg L^−1^ (light field and fluorescence field). (**a–e**) Clear field and (**f–j**) fluorescent field. *Bar* = 20 µm.

Analysis by multiphoton microscopy allowed us to observe the presence of AgNPs in epidermal stevia leaf and stem cells. The results show the presence of AgNPs in epidermal cells of the stem cross-section in the different treatments with NPs (Fig. 5b,c). However, as the concentration of AgNPs increases at 100 and 200 mg L^1^, they are more frequently observed in the intercellular spaces of epidermal cells (Fig. 5d,e). The sequence of images in Fig. 5 shows the clear field (a-e) and fluorescence of the AgNPs (f-j) and the progression of fluorescence in the stem cross-section in different treatments with AgNPs.

**Figure 6:**
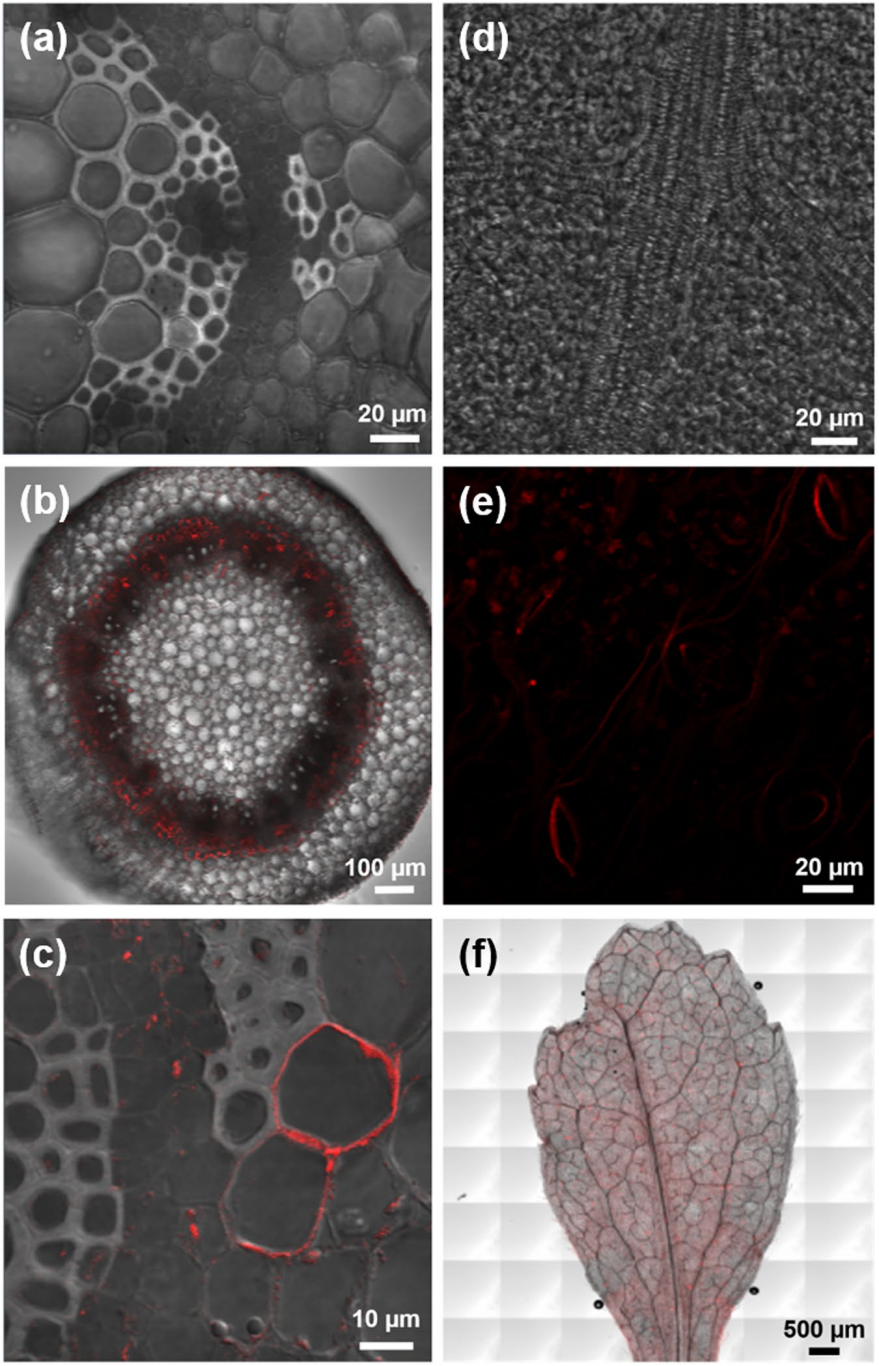
Identification of fluorescence by a multiphoton microscope at specific points on the stem and stevia leaf. (**a**) Vascular beams of the control treatment, (**b**) cross-section scan of stem with 100 mg L^−1^, (**c**) presence of silver in cells with 50 mg L^−1^, (**d**) leaf rib control, (**e**) fluorescence on stomatas at 25 mg L^−1^ (**f**) fluorescence in leaf at 200 mg L^−1^.

The tissue stem with 200 mg L ^−1^ shows a higher incidence of fluorescence in the stem tissue (Fig. 5e). No fluorescence was found in the stem, xylem and phloem cells of the control treatment (Fig. 6a). However, in all AgNP-treated stevia stems it was possible to observe fluorescence within vascular bundles and in nearby cells (Fig. 6b). Fluorescence emitted by AgNPs was observed around the cells of the cortical parenchyma, having great affinity for the intercellular spaces (Fig. 6c). In the case of leaves, the control treatment without AgNPs did not show fluorescence emitted by AgNPs (Fig. 6d), while in leaves treated with AgNPs fluorescence was found in epidermal leaf and stoma cells (Fig. 6e). Finally, scanning of the entire surface of the AgNP-treated leaf shows a possible internalization into foliar tissues as concentrations of AgNPs increase. In addition, the propagation of the middle vein fluorescence signal to the mesophilic and parenchymal tissues was observed (Fig. 6f).

## Discussion

In this study, the TEM results allowed us to characterize the shape and size of Argovit. It is known that spherical silver particles do not emit fluorescence in the red spectrum, as their dispersion properties can be adapted according to their size, shape and composition^15^. When obtaining fluorescence peaks at 427, 514 and 631 nm, it is suggested that various AgNP sizes and shapes are found in the Argovit solution. In addition, smaller AgNPs have been reported to be more toxic to plants^16^. According to^17^ and^18^, the toxicity of AgNPs depends on the size, shape, coating and concentration of Ag+ added.

Adding AgNPs to the culture medium significantly affected shoot multiplication and length. The lowest AgNP concentrations promoted the greatest shoot production and length per explant. Similar results were obtained by^19^ in seedlings of wheat (*Triticum aestuvum*) cv. NARC-2009 grown *in vitro* in MS medium with different concentrations, finding better development in 25 mg L^−1^ of AgNPs. In vanilla (*Vanilla planifolia*)^7^, using AgNPs in automated temporary immersion vessels (RITA^®^) observed increased shoot production and length at a concentration of 50 mg L^−1^ of AgNPs. Recently^8^, in sugarcane (*Saccharum* spp.) using the Temporary Immersion Bioreactor (BIT^®^) reported a hormetic effect when using AgNPs at low concentrations, finding that concentrations above 50 mg L^−1^ cause the production of reactive oxygen species (ROS). Activation of the antioxidant response from plant exposure to metallic NPs has already been used to promote positive effects on callus induction, shoot regeneration and *in vitro* growth^5^.

In rice (*Oryza sativa* L.)^20^, reported an increase in ROS in roots of seedlings exposed to AgNPs. According to^21^, this effect is due to the fact that low concentrations of heavy metals induce hormetic effects through the activation of plant stress defense mechanisms. On the other hand^22^, mentions that toxic metals directly or indirectly trigger the generation of ROS, acting as signals that stimulate the activation of genes during the detoxification of ROS. In our study, from a dose of 50 mg L^−1^ the dry matter increased, which coincides with the findings of^23^, who point out that AgNPs at 25 and 50 mg L^−1^ have the capacity to significantly increase plant height and both fresh and dry weight in wheat.

The results obtained for chlorophyll contents coincide with those reported by^19^, who in *in vitro*-grown wheat found that total chlorophyll increased significantly at concentrations of 25, 50 and 100 mg L^−1^ of AgNPs. On the other hand^24^, observed in beans (*Phaseolus vulgaris*) and corn (*Zea mays*) that applying 60 mg L^−1^ of AgNPs promoted growth and chlorophyll content^23^, in mung bean (*Vigda radiata*), observed an increase in chlorophyll contents at concentrations of 50 mg L^−1^ of AgNPs^20^. Demonstrated that exposure of rice (*Oryza sativa*) to 0.5 mg L^−1^ of AgNPs not only significantly decreases root and shoot biomass, but also increases the content of chlorophyll and carotenoids. Recently^7^, and^8^ reported an increase in the content of photosynthetic pigments in vanilla and sugarcane shoots treated with AgNPs, this effect was probably due to the increase in N, Mg and Fe concentrations in plant tissues exposed to AgNPs, since these elements are associated with chlorophyll biosynthesis. N is essential in molecules such as chlorophyll, proteins and nucleic acids, while Mg is necessary for reactions involving adenosine triphosphate and is found in the porphyrin moiety of the chlorophyll molecule, while Fe is important in the catalytic group for redox enzymes^25,26^, reported that the use of NPs in plants alters photochemical fluorescence, photosynthetic efficiency and quantum yield. Some authors have reported that AgNPs improved the quantum efficiency of photosystem I in green algae (*Chlamydomonas reinhardtii*)^27^ and photosystem II in brown mustard (*Brassica juncea*)^28^. Thanks to this, knowledge about the interactions of AgNPs with photosynthetic machinery provides knowledge about the oxidative stress induced by AgNPs and the antioxidant defense system in plants. Therefore, it is possible that the results of our study demonstrate that the defense mechanisms of stevia plants were activated by the addition of different AgNP concentrations.

The bioaccumulation of Ag+ has been previously studied in plant systems^29^. exposed lettuce (*Lactuca sativa*) plants to 100 mg L^−1^ of AgNPs and found that the leaves had 18.93 μg g^−1^ dw of Ag+^4^. used a foliar spray to apply AgNPs to two varieties of beans and found that by adding 60 mg L^−1^ of AgNPs they obtained 0.35 and 0.49 μg g^−1^ dw of Ag+ in whole plants of the Bronco and Nebraska varieties, respectively, compared to 0.25 and 0.29 μg g^−1^ dw in the controls. In our stevia control plants, we found a lower amount of Ag+ contained in the dry tissue with 0.13 μg g^−1^ dw^30^. analyzed root and shoot samples of *Arabidopsis thaliana*, with foliar application of 75 and 300 mg L^−1^ of AgNPs, finding that the roots accumulated 10 times more Ag+ than the shoots. In our study, the highest Ag+ concentrations in foliar tissue (95.23 and 188.16 μg g^−1^ dw) were found at high concentrations of AgNPs (100 and 200 mg L^−1^ of AgNPs). However, it was not possible to analyze root samples, which opens up the possibility of studying the levels of Ag accumulation in whole stevia plants, and even to continue to evaluate the accumulation of Ag in stevia tissues under *ex vitro* conditions. The results obtained in this research on the bioaccumulation of Ag in stevia tissues *in vitro* indicate the need for future studies on genotoxicity, karyotypes and the presence of micronuclei.

It is difficult to compare studies and interpret some of the work done *in vitro* as each study involves NPs of different sizes, shapes, ion concentrations and types of surface coatings. Although it is possible to find more studies on the location of AgNPs in roots^16,19^ in this study we focus on locating AgNPs in stem and leaf tissues of stevia *in vitro* using fluorescence microscopy techniques. The rinsing technique developed for this work allowed the chlorophyll present in foliar tissues and pigments associated with epidermal stem cells to be extracted, eliminating autofluorescence and allowing the signal from the excitation of the AgNPs to be recovered.

Optical and fluorescence microscopy has already been used as a tool in the study of the internalization mechanism of nanomaterials in plants^31,32^. The same technology has been used to locate AgNPs in plant tissues of *Arabidopsis thaliana in vitro*^30^ and in lettuce leaves^29^. In the images obtained as evidence in this study, it was possible to observe that the tissue exposed to different AgNP doses presents greater fluorescence induced in the epidermis and supports the results mentioned above. In addition, it is shown that the amount of fluorescence increases in proportion to the concentration used in our treatments. According to^33^, nanomaterials can be transported from the root, through the extracellular spaces of the cells (apoplastic pathway) to the endodermis or vascular system, with the xylem being the most important vehicle in the distribution and translocation of NPs. However, to cross the cell membrane, NPs are transported through the pores, indicating that the uptake of nanomaterials is size specific^26^.

In our study, it was possible to observe small points of fluorescence inside the cells, probably due to the fact that smaller NPs cross the pores of the membrane, considering that, according to^34^, the pores of the cell membranes oscillate between 3.5–5.5 nm. However, nanomaterials can also be transported through plasmodesmata, channels of approximately 40 nm in diameter that connect adjacent cells^35^. According to our characterization, the size of Argovit NPs is 35 ± 15 nm, which indicates that our NPs are the ideal size to be transported by plasmodesmata. Several reports have explored the transport and accumulation mechanisms of NPs in plant tissues. On the other hand^36^, mention that nanometric-size salts can enter leaf tissue through two mechanisms: through the cuticle or through stomatal pores. However, for the most part, the studies that characterize the transport of AgNPs have been based on hydroponic systems, with few studies on *in vitro* plants^29^. found Ag agglomerated on the surface of the leaves, mesophyll, epidermis, vascular bundles and in stomata. This coincides with what was observed in stevia leaves, showing that the fluorescence emitted by the AgNPs allowed their visibility on leaf and stoma surfaces under *in vitro* conditions.

## Methods

### Silver Nanoparticles characterization

#### Silver nanoparticles formulation

The AgNPs used in this study, formulated as Argovit^®^, were provided by the Production Centre Vector-Vita Ltd, located in Novosibirsk, Russia. Argovit is made up of 12 mg mL^−1^ of metallic silver and 188 mg mL^−1^ of polyvinylpyrrolidone (PVP, 15–30 kD) (20%). Characterization of physicochemical properties was performed from a fraction of Argovit AgNPs, which was precipitated by centrifugation at 11,000 rpm for 10 min. Afterwards the PVP was removed and resuspended with 70% acetone. This washing cycle was performed three times and in the last step it was resuspended with deionized water.

#### Electron microscopy characterization

Morphological and morphometric characterization of AgNPs was performed using a transmission electron microscope (Morgagni M-268, Philips/FEI, The Netherlands). For the morphological study, 5 μL of NPs were deposited on a 300 mesh formvar/carbon coated copper grid (Electron Microscopy Science, PA). The sample was incubated for 10 minutes and the excess was removed, after which it was dried at room temperature. The operating conditions of the microscope were: high magnification (140,000X) at high voltage 80 kV extra high voltage (EHT) with the column pressure of 5 × 10^−3^ Pa (5 × 10^−5^ Torr). All micrographs were captured in TIFF format with a size of 1376 × 1032 pixels in grayscale. In this format, 0 corresponds to black pixels and 255 to white. The morphometric descriptors evaluated were circularity, shape factor, solidity and effective diameter^37^.

#### Plant material and *in vitro* establishment

Nodal segments of 3 cm in length of stevia (*Stevia rebaudiana* B.) cv. Morita II were collected from the greenhouse. The explants were disinfected in a surfactant solution (two drops of Tween-20 in 1 L of distilled water) and washed with a slow flow of running water for 30 minutes. Subsequently, in a laminar flow hood they were immersed in 70% (v/v) ethanol for 30 s and in 0.6% and 0.3% (v/v) sodium hypochlorite for 10 and 5 min, respectively. Three rinses with sterile water were performed. Finally, the explants were planted in test tubes. Each tube contained 10 mL of MS^38^ medium supplemented with 2 mg L^−1^ of 6-Benzylaminopurine (BAP) with 2.5 g L^−1^ of Phytagel^®^ added as the gelling agent. The culture medium was sterilized at 120 °C at 115 kPa for 20 min. The incubation period was 24 ± 2 °C and they were kept under fluorescent light (40–50 μmol m^−2^ s^−1^) with a photoperiod of 16 h.

#### Effect of AgNPs during shoot multiplication

After two subcultures in semi-solid media, 10 explants were placed in Temporary Immersion Bioreactors (TIB^®^^39^,) with a volume of 1 L, containing 500 mL of liquid MS medium supplemented with 2 mg L^−1^ of BAP. After sterilizing the medium, different concentrations of AgNPs (0, 12.5, 25, 50, 100 and 200 mg L^−1^) were added using three TIBs per treatment. The entire experiment was conducted in triplicate. At 30 days of culture, the number of shoots per explant, shoot length per explant and dry matter were recorded. The dry matter content was calculated using dry weight/fresh weight × 100. The dry weight was determined after placing the stevia shoots in a drying oven at a temperature of 65 °C for 72 h.

#### Chlorophyll content

The content of chlorophyll a (chl a), chlorophyll b (chl b) and total chlorophyll (total chl) was determined in leaves according to the methodology proposed by^40^. For each treatment, 0.2 g of fresh matter were weighed and then macerated with a mortar using 80% acetone. The samples were taken to a temperature of −4 °C for 24 h. The mixture was adjusted to 6.25 mL with 80% acetone and filtered with Whatman No. 41 paper. Finally, absorbance readings were made using a Spectrophotometer at 663 nm (chl a) and 645 nm (chl b). Chlorophyll a, b and total contents were calculated using the following equations:

$$\begin{array}{rcl}Chlorophyll\,a\,(C) & = & \frac{[(12.7\ast {A}_{663})-(2.5\ast {A}_{645})](V)}{(1000\ast P)}\\ Chlorophyll\,b\,(C) & = & \frac{[(22.9\ast {A}_{645})-(4.70\ast {A}_{663})](V)}{(1000\ast P)}\\ Total\,Chlorophyll\,(C) & = & Chlorophyll\,a+Chlorophyll\,b\end{array}$$

Where:

A = Absorbance.

C = Concentration (mg g^−1^ fresh weight).

V = Volume (m L^−1^).

P = Sample weight (g).

1000 = Conversion factor.

#### Measurement of silver, magnesium, nitrogen and iron content

Measurements were determined using Inductively Coupled Plasma-Mass Spectrometry (ICP-MS). Fresh matter shoots were taken from the different treatments with AgNPs. The fresh tissue samples were washed with running water for 15 min and placed in a drying oven at a temperature of 65 °C for 72 h following the protocol described by^41^. Then 250 mg of dry tissue were weighed per sample and immersed in a solution of H_2_SO_4_:HClO_4_ (2:1, v:v). Subsequently, the samples were taken to a volume of 25 mL with deionized water. Finally, the samples were filtered and the digestion extracts were read by ICP-MS (Varian ICP OES 725-ES; Mulgrave, Australia).

### Evaluation of NPs deposition in tissues

#### Multiphoton microscopy

The detection of the emission spectrum of the AgNPs and the visualization of the deposition in stevia tissues was performed by a hybrid multiphoton microscopy system (Axio Imager Z2, LSM 880-NLO, Zeiss, Germany) coupled to a Ti:Sapphire infrared laser (Chameleon Vision II, COHERENT, Scotland) with tuning capability in the range of 690 to 1060 nm. Operating conditions in all experiments involved using the Chameleon laser at 900 nm with 1.2% power, pinhole at 601.1 and photodetector voltage ranges in similar conditions. Leaf and stem reconstructions were performed with a 20X/0.5 objective, NA ∞−0.17, Zeiss Plan NEOFLUAR and observations at high magnifications were performed using a 63x/1.3 immersion objective, NA ∞−0.17, Zeiss Plan NEOFLUAR. The specific fluorescence of the AgNPs was obtained through the “Lambda” scanning method allowing the detection of photons in the emission spectrum from 398 to 719 nm, with a spectral sensitivity of 5 nm. All micrographs were captured in CZI format in a size of 1131 × 1131 pixels composed of three color channels (RGB).

#### Fluorescence microscopy

The identification of AgNPs was performed on stevia leaves and stems (without roots). Internodal stem segments were dissected and cross-sections were made with the free hand technique, while complete samples were taken in the case of leaves; both tissues were fixed in an FAA solution (formaldehyde, acetic acid, ethanol-water) and incubated for 24 hours. The samples were then washed three times with phosphate buffer solution at 0.16 M, pH 7.4. The samples were placed in 50% ethanol for 3 hours. After this treatment, they were immersed in ethanol, water and glycerin (1:1:2) for 3 hours and placed in ethanol and glycerin (1:1) for 12 hours. Finally, to remove all chlorophyll, three washes were performed with 70%, 80% and 100% ethanol during one hour of incubation and rinsed with phosphate buffer solution and deionized water. The stem and leaf cross-sections were fixed on a slide with deionized water and covered with high-transparency cover glasses (“Zeiss cover glasses” D = 0.17 mm +/− 0.005 mm, refractive index = 1.5255 +/− 0.0015, Abbe Number = 56 +/−2). The same scanning conditions described in the multiphoton microscopy section were used to determine the fluorescence emitted by the AgNPs.

#### Statistical analyses

A completely randomized experimental design was used. Statistical analysis was performed by one-way ANOVA and comparison of means according to Tukey (*P* ≤ 0.05) using SPSS v. 22 for Windows. Arcsine transformation was performed for experimental data taken in percentages before submitting them to statistical analysis.

## Conclusions

The administration of AgNPs (Argovit) in stevia explants established *in vitro* affects their development and chlorophyll content. It was clear that they cause hormetic effects in stevia, since exposure to low concentrations of AgNPs stimulates growth while increasing the concentration of AgNPs inhibits development. The content of Ag retained in stevia shoots depends on the Argovit concentrations added to the culture medium.

High AgNP doses mostly bioaccumulate in stevia cells and tissues. Multiphoton microscopy allowed these accumulation patterns to be established through an internalization pathway that passes through the vascular bundles and are translocated to neighboring cells via the apoplast, forming a gradient of particle aggregates that are mostly deposited between the intercellular spaces and reach leaves where they can be deposited in stomata, probably following the flow of water and nutrients that diffuse through cellular communication. Probably high concentrations of AgNPs cause genocitotoxic damage because they are internalized in influencing the cellular mechanisms of cell division and repair.

The use of AgNPs has been shown to have a positive effect on shoot production and length, as a microbicidal agent, as an inhibitor in ethylene synthesis, in photosynthetic pigment synthesis, nutrient accumulation, antioxidant metabolism and ROS generation. Our study contributes knowledge to improve our understanding of the effects and transport, localization and translocation mechanism of NPs applied to the *in vitro* culture of plant tissues. In addition, our results open up the possibility of applying Argovit to evaluate its use potential in *in vitro* crops of agricultural interest.
